# Multidrug-resistant bacterial isolates in infected wounds at Jimma University Specialized Hospital, Ethiopia

**DOI:** 10.1186/1476-0711-12-17

**Published:** 2013-07-23

**Authors:** Girma Godebo, Gebre Kibru, Himanot Tassew

**Affiliations:** 1 School of Medicin, Wachemo University, Hosaena, Ethiopia; 2 Department of Medical Laboratory Sciences and Pathology, Jimma University, P. O .Box 196, Jimma, Ethiopia

**Keywords:** Multi drug resistance, Wound infection, Jimma, Ethiopia

## Abstract

**Background:**

The term ’Multidrug-resistant’ (MDR) applies to a bacterium that is simultaneously resistant to a number of antimicrobials belonging to different chemical classes. The effectiveness of currently available antmicrobial drugs is decreasing due to the increasing number of resistant strains causing infections so that available therapeutic options for such organisms are severely limited.

**Objective:**

The aim of this study was to determine multidrug-resistance rate of bacterial isolates that caused wound infections.

**Methods:**

A Hospital based cross-sectional study was conducted on 322 wound samples taken from consecutive patients seen at inpatient and outpatient department of Jimma University Specialized Hospital from June to December 2011. Swabs from surgical incisions, burns, abscess and traumatic wounds were collected aseptically using Levine’s technique. Bacteriological culture and examination was done following standard microbiological techniques. Multidrug-resistance test was performed by disk diffusion method against 10 classes of antimicrobials. The data was analyzed for descriptive statistics using SPSS version 16 and Microsoft Excel.

**Results:**

The overall MDR among gram positive and gram negative bacterial isolates were (77%) and (59.3%) respectively. About, 86.2% *S.aureus* and 28.6% of Coagulase negative *Staphylococci* became MDR. Nearly 30.1% of *S.aureus* was resistant to six classes of antimicrobials. The average MDR rate of *Proteus*, *Klebsiella*, and *Providencia* species was 74.8%, 69.6% and 75% in that order. Nearly, 30.8% of *Proteus sp*, 32.6% of *Klebsiella* sp and 61% of *Citrobacter* sp were resistance to 4 classes each. Surprisingly, the average MDR rate for *Citrobacter* sp was 100%. About (76.7%) of *S.aureus* was Oxacillin/Methicillin resistant while (16.4%) were Vancomycin resistant. *Proteus* species was the predominant isolates (27.9%) followed by *P.aeruginosa* and *S.aureus* (19.3%) and (19%) respectively.

**Conclusion:**

This study indicated that, the overall rate of MDR bacterial pathogens that caused wound infection was very high and many of the isolates were also identified as resistant to three or more classes of antimicrobials. Such widespread resistance to antimicrobial classes is something serious because a few treatment options remain for patients with wound infections. Periodic monitoring of etiology and antimicrobial susceptibility in areas where there is no culture facility is essential to assists physician in selection of chemotherapy.

## Introduction

The term multidrug-resistant (MDR) applies to a bacterium that is simultaneously resistant to a number of antimicrobial drugs belonging to different chemical classes or subclasses through various mechanisms [1]. One of the methods used by many authors and authorities to characterize organisms as MDR is based on the results of invitro antimicrobial susceptibility test. Antimicrobial drug resistance can be acquired as a result of mutation or acquisition of resistance genes via horizontal gene transfer, or can be an innate feature of an organism that is encoded chromosomally [2].

MDR in both the hospital and community environment are important concern to the clinician, patients and the pharmaceutical industries [3]. The widespread uses of antibiotics, together with the length of time over which the drugs have been available at market have led to major problems of the emergence of resistant organisms [4]. Antimicrobial drugs overuse, over dosing, drugs prescription with improper susceptibility test, self-medication and long duration of hospitalization was suggested to augment the problem of MDR in developing nations [5]. The Centers for Disease Control and Prevention (CDC) report regarding healthcare associated infection indicated that antimicrobial-resistant gram-negative bacilli are an emerging threat in the healthcare setting [6].

The study conducted on MDR among gram-negative pathogens that caused healthcare-associated infections in Atlanta, Georgia (USA) evaluated that 10% of *P.aeruginosa*, and 15% *K.pneumoniae* were found to be both resistant to 3 antimicrobial class. A much larger proportion, 60% of *Acinetobacter baumannii* isolates were resistant to at least 3 antimicrobial classes. Though less common than 3-class resistance, isolates with 4-class resistance were also seen in significant numbers and across regions [7].

Zeleke WT [8,9] in his part indicated that over the past few years several studies in African countries had reported the presence of MDR strains of bacteria identified from clinical and environmental specimens. This was consecutively ascertained by findings of Olayinka *et al*., 2004 [10], Chikere *et al.*, 2008 [11], Nkang *et al*., 2009 [5] all in Nigeria, Zeleke, 2002 in Ethiopia [8] and Anguzu *et al.*, 2003 in Uganda [12]. A study conducted in one of the tertiary hospitals in Ethiopia also reported that about 51% of the gram negative bacterial isolates from open wounds were identified as MDR [13].

Such increase in both community and hospital-acquired antimicrobial-resistant bacteria is challenging the ability of effective patient treatment, prudent infection control and new treatment alternatives. According to Olayinka *et al.,* constant bacteriological monitoring of the pathogens isolated from clinical specimens of patients in special units is necessary to draw attention of clinicians and infection control specialists to their current antibiotic susceptibility pattern and how often specific pathogens are isolated [10]. Blomberg and his colleague also suggested that the widely emerging MDR pathogens, in the absence of appropriate antimicrobial resistance surveillances and organized prevention strategies added worries in the incidence of infections among surgically operated, burn and other traumatic wound patients [14].

Though several studies have been conducted on etiology of wound infections in Ethiopian, none of them adequately addressed the extent of drug resistance of these isolates against different antimicrobial classes. Therefore, this study was intended to determine the magnitude of MDR bacteria identified from infected wounds in order to provide locally applicable data and to guide empirical therapy in area where culture and drug susceptibility testing facilities are scarce.

## Methods

### Study design and population

This facility based cross sectional study was conducted in Jimma University Specialized Hospital (JUSH) from May to December 2011. It is a 300 bedded hospital covers more than one million people living in the western regions of the country and gives specialty services in 11 wards and up to 400 patients attending outpatient department daily. Sociodemographic and clinical data of participant patients was obtained using semi-structured questionnaire.

### Wound sample collection and processing

During the study period, a total of 322 infected wound samples were collected from consecutive patients seen both inpatient and outpatient departments. Wound beds were prepared before specimen collection by using Levine’s technique [15], where the wound immediate surface exudates and contaminants were cleansed off with moistened sterile gauze and sterile normal saline solution. Dressed wounds were cleansed with non bacteriostatic sterile normal saline after removing the dressing. This technique is believed to be the best technique for swabbing open wounds and more reflective of tissue bioburden than swabs of exudates or swabs by other techniques. Cleansing the wounds prior to obtaining swab specimens was done in an effort to remove immediate surface contaminating organisms (bacteria). Thus the culture will be more likely to represent the microbiology in the deep wound compartment [15,16]. Aseptically the end of a sterile cotton-tipped applicator was rotated over 1 cm^2^ area for 5 seconds with sufficient pressure to express fluid and bacteria to surface from within the wound tissue as technique stated by Levine *et al.,* 1976 [15] and Gardner *et al.,* 2007 [16]. Double wound swabs were taken from each wound at a point in time to reduce the chance of occurrence of false-negative cultures and to increase the chance of recovering bacterial pathogens. It is also indicative of contamination in that if the two swab samples differ in types of organisms during presumptive test [17]. Then, wound specimens were transported to microbiology laboratory within 20 minutes by placing the swabs in to the sterile test tubes having 0.5 ml of sterile normal saline solution. Bacteriological culture and examination was done following standard microbiological techniques [18].

### Multidrug-resistance testing

Multidrug-resistance test was performed by disk diffusion method according to the criteria set by the (CLSI, 2010) [19] against different classes of antimicrobials: Cephalosporin class (cefoxitin, cefotaxim, ceftriaxone); Aminoglycosides class (gentamycin); Fluorquinolones class (ciprofloxacin, norfloxacin), Tetracycline class (doxycyclin); Folate Pathway Inhibitors (cotrimoxazole); Phenicols class (chloramphenicol); Penicillin class (oxacillin, ampicllin, penicillin); Glycopeptides class (vancomycin); Macrolides class (erythromycin) and Lincosamides class (clindamycin). Cloxacillin is not classified as individual class of drug rather it is classified as related drug to penicillinase resistant penicillin group like oxacillin and dicloxacillin. Gram positive bacteria were tested for drugs selected from all ten classes of antimicrobials where as gram negative were tested for seven classes excluding Glycopeptides, Macrolides and Lincosamides. The antimicrobial disks used for the test were all from (Oxoid Ltd. England). These drugs were selected based on the national list of medicines (FMHACA Ethiopia, 2010) to treat infections, prescription frequencies and availability. In order to monitor quality (potency) of disks, a standard strain of *P.aeruginosa* (ATCC-27853), *S.aureus* (ATCC-25923) and *E.coli* (ATCC-25922) were tested at regular interval and whenever new batches of antimicrobial discs were used.

### Data analysis

The data was analyzed for descriptive statistics using SPSS version 16 and Microsoft Excel and presented in forms of tables. The results were interpreted in terms of frequencies, and percentages.

### Ethics

This study was conducted after obtaining separate permission from Jimma University Ethical review Board and the Management Committee of Jimma University Specialized Hospital. Informed consent was also pursued from patients or guardians of children and any information was kept confidential. All laboratory tests were done free of charge and their results were communicated to patients respective physician or nurses for beneficiary measures.

## Results

### Multidrug-resistant patterns of isolates

In this study, multidrug-resistant (MDR) status of gram positive and gram negative bacteria was tested against 10 and 7 classes of antimicrobials respectively. Accordingly, the overall rate of MDR among gram positive isolates was 77%. This means, 86.2% of *S.aureus* and 28.6% of Coagulase negative *Staphylococci* (CNS) were becoming MDR. Moreover, 30.1% of *S.aureus* showed resistance to six antimicrobial classes. About 21.4% of CNS was resistant to three classes as well (Table 1).

**Table 1:** MDR gram positive bacteria identified from infected wounds in JUSH, June to December 2011

**Bacteria**	**Antimicrobial classes resisted to No (%)**								**Average**
	**R3**	**R4**	**R5**	**R6**	**R7**	**R8**	**R9**	**R10**	
**S. aureus**	2(2.7)	6(8.2)	11(15.1)	22(30.1)	11(15.1)	4(5.5)	2(2.7)	5(6.9)	**63(86.2)**
**CNS**	3(21.4)	0(0.0)	1(7.1)	0(0.0)	0(0.0)	0(0.0)	0(0.0)	0(0.0)	**4(28.6)**
**Total**	5(5.8)	6(6.9)	12(13.8)	22(25.3)	11(12.6)	4(4.6)	2(2.3)	5(5.8)	**67(77.0)**

Key: R3 - R10 = resistance of bacteria to 3,4,5,6,7,8,9 or 10 classes of antimicrobials tested.

Then again, the overall MDR rate of gram negative bacteria was 59.3%. Relatively higher rate of MDR was seen among *Proteus*, *Klebsiella* and *Providencia* species accounting average resistance of 74.8%, 69.6% and 75% respectively. Additionally, 24.3% of *Proteus* and 25% of *Providencia* species were resistant to three classes. About 32.6% of *Klebsiella* sp also showed resistant to four classes. Surprisingly, the average MDR rate of *Citrobacter* sp was found out to be 100% (Table 2).

**Table 2:** MDR gram negative bacteria identified from infected wounds in JUSH, June to December 2011

**Bacteria**	**Classes of antimicrobial resisted to No (%)**					**Average No (%)**
	**R3**	**R4**	**R5**	**R6**	**R7**	
***Proteus spp***	26(24.3)	33(30.8)	12(11.2)	5(4.8)	4(3.7)	**80(74.8)**
***P. aeruginosa***	11(14.9)	9(12.2)	3(4.1)	-	4(5.4)	**27(36.5)**
***Klebsiella spp.***	3(6.5)	15(32.6)	1(2.2)	-	13(28.3)	**32(69.6)**
***E. coli***	3(10.0)	3(10.0)	1(3.3)	-	-	**7(23.3)**
***Citrobacter***	1(5.5)	11(61.0)	1(5.6)	3(16.7)	2(11.1)	**18(100)**
***Providencia spp.***	2(25.0)	1(12.5)	2(25.0)	-	1(12.5)	**6(75.0)**
***Acinetobacter spp.***	-	2(28.6)	1(14.3)	-	1(14.3)	**4(57.2)**
***M. morganii***	-	1(20.0)	1(20.0)	-	-	**2(40.0)**
**Total**	46(15.5)	75(25.2)	22(7.4)	8(2.7)	25(8.4)	**176(59.3)**

Key: R3- R7 = resistant to 3, 4, 5, 6 or 7 classes of antimicrobials tested.

### Antimicrobial resistance pattern to individual drugs

The drug resistance profile of gram positive bacterial isolates tested for 16 antimicrobials showed that 94.5% of *S.aureus* was resistant to penicillin, 91.8% to ampicillin and 76.7% to oxacillin. About 16.4% of *S.aureus* became vancomycin resistant. Similarly, 68.3% of coagulase negative *Staphylococcus* (CNS) was resistance to both penicillin and ampicillin. Fortunately, CNS was 100% sensitive to many of the antimicrobial drugs tested (Table 3).

**Table 3:** Antimicrobial drugs resistance pattern of gram positive bacteria identified from infected wounds in JUSH, June to December 2011

**Bacteria**	**Drugs No (%) resistance to**																**Total (%)**
	**OX**	**FOX**	**OB**	**E**	**CD**	**P**	**AP**	**DO**	**CRO**	**NOR**	**CTX**	**TS**	**CN**	**VA**	**CIP**	**C**	
***S .aureus *(n = 73)**	56 (76.7)	21 (28.8)	57 (78.1)	58 (79.4)	62 (85.0)	69 (94.5)	67 (91.8)	60 (82.2)	15 (20.5)	12 (16.4)	26 (35.6)	44 (60.3)	12 (16.5)	12 (16.4)	10 (13.7)	51 (69.9)	**(54.1)**
***CNS *(n = 14)**	1 (7.1)	0 (0.0)	3 (21.4)	0 (0.0)	0 (0.0)	9 (68.3)	9 (68.3)	5 (35.7)	2 (14.3)	0 (0.0)	2 (14.3)	5 (35.7)	0 (0.0)	0 (0.0)	0 (0.0)	4 (28.6)	**(17.9)**

Key: OX = Oxacillin, FOX = Cefoxitin, OB = Cloxacillin, E = Erythromycin, CD = Clindamycin, P = Penicillin, AP = Ampicillin, DO = Doxycycline, CRO = Ceftriaxone, NOR = Norfloxacin, CTX = Cefotaxim, TS = Cotrimoxazole, CN = Gentamycin, VA = Vancomycin, CIP = Ciprofloxacin, C = Chloramphenicol.

On the other hand, the resistance patterns of gram-negative bacteria isolates (n = 297) tested against nine antimicrobial drugs showed that *P.aeruginosa* was 97.3%, 87.8%, and 83.8% resistance to ampicillin, cotrimoxazole, and doxycycline respectively. Similarly, *Citrobacter* species showed 100% resistance to ampicillin, cotrimoxazole and chloramphenicol and 88.9% to doxycycline. Furthermore, *Proteus* species showed 85% resistance to chloramphenicol and 75.7% to cotrimoxazole. With the exception of *Citrobacter* and *Proteus* sp, all other gram negative isolates in this study showed relatively low resistance to ceftriaxone, cefotaxim, norfloxacin, ciprofloxacin and chloramphenicol (Table 4).

**Table 4:** Antimicrobial drug resistance patterns of gram negative bacteria identified from infected wounds in JUSH, June to December, 2011

**Bacteria**	**Drugs resisted to No (%)**									**Average (%)**
	**AP**	**DO**	**CRO**	**CTX**	**NOR**	**CIP**	**CN**	**TS**	**C**	
***Proteus spp. *(n = 107)**	77(72.0)	80(74.8)	8(7.5)	14(13.1)	6(5.6)	8(7.5)	35(32.7)	81(75.7)	91(85.0)	**(39.9)**
***P.aeruginosa *(n = 74)**	72(97.3)	62(83.8)	7(9.5)	9(12.2)	5(6.8)	4(5.4)	8(10.8)	65(87.9)	55(74.3)	**(43.5)**
***Klebsiella spp. *(n = 46)**	32(69.6)	36(78.3)	13(28.3)	14(30.4)	13(28.3)	13(28.3)	13(28.3)	30(65.1)	32(69.6)	**(47.3)**
***E. coli *(n = 30)**	23(76.7)	7(23.3)	20(66.7)	20(66.7)	1(3.3)	1(3.3)	0(0.0)	6(20.0)	4(13.3)	**(30.4)**
***Citrobacter *spp (n = 18)**	18(100)	16(88.9)	3(16.7)	5(27.8)	2(11.1)	2(11.1)	6(33.3)	18(100)	18(100)	**(54.3)**
***Providencia *spp (n = 8)**	7(87.5)	4(50.0)	2(25.0)	3(37.5)	1(12.5)	1(12.5)	2(25.0)	6(75.0)	7(87.5)	**(45.8)**
***Acinetobacter spp *(n = 7)**	4(57.1)	3(42.9)	4(57.1)	4(57.1)	1(14.3)	1(14.3)	3(42.9)	3(42.9)	4(57.1)	**(42.9)**
***M. morganii *(n = 5)**	5(100)	5(100)	0(0.0)	1(20.0)	0(0.0)	0(0.0)	0(0.0)	2(40)	2(40.0)	**(33.4)**
***E. cloacae *(n = 2)**	0(0.0)	0(0.0)	0(0.0)	0(0.0)	0(0.0)	0(0.0)	0(0.0)	0(0.0)	0(0.0)	**0(0.0)**

Key: AP = Ampicllin, DO = Doxycyclin, CRO = Ceftriaxone, NOR = Norfloxacin, CTX = Cefotaxim, TS = Cotrimoxazole, CN = Gentamycin, CIP = Ciprofloxacin, C = Chloramphenico.

### Etiology of wounds

In this study, 96.3% of wound samples were culture positive of which 22.9% had multiple bacterial infections (data not shown). As it is indicated in Table 5, the most prevalent wound type was trauma (37.8%) followed by abscess (29.8%) and the least was cellulites (1%). *Proteus* species was the most frequently isolated bacteria accounting 27.9% followed by *P. aeruginosa* and *S. aureus* with rate of 19.3% and 19% respectively.

**Table 5:** Frequency of pathogenic bacteria isolates by wound types at JUSH from June to December 2011

**Bacteria**	**Wound type No (%)**						**Total**
	**Surgical**	**Abscess**	**Trauma**	**Burn**	**Osteomyelitis**	**Cellulites**	
*Proteus sp*	19 (4.9)	45(11.7)	26(6.8)	13(3.4)	3(0.9)	1(0.3)	107(27.9)
*P.aeruginosa*	8(2.1)	11(2.9)	45(11.7)	8(2.1)	2(0.5)	0(0.0)	74(19.3)
*S.aureus*	15(3.9)	23(6.0)	23(6.0)	7(1.8)	3(0.9)	2(0.5)	73(19.0)
*Klebsiella sp*	1(0.3)	1(0.3)	30(7.8)	14(3.6)	0(0.0)	0(0.0)	46(12.0)
*E.coli*	3(0.9)	20(5.2)	5(1.3)	2(0.5)	0(0.0)	0(0.0)	30(7.9)
*Citrobacter sp*	5(1.3)	5(1.3)	4(1.0)	4(1.0)	0(0.0)	0(0.0)	18(4.7)
*CNS*	2(0.5)	6(1.6)	5(1.3)	1(0.3)	0(0.0)	0(0.0)	14(3.6)
*Providencia sp*	2(0.5)	3(0.8)	0(0.0)	0(0.0)	0(0.0)	0(0.0)	8(2.1)
*Acinetobacter*	1(0.3)	1(0.3)	1(0.3)	2(0.5)	1(0.3)	1(0.3)	7(1.8)
*M.morganii*	3(0.8)	0(0.0)	1(0.3)	0(0.0)	1(0.3)	0(0.0)	5(1.3)
*E.cloacae*	0(0.0)	0(0.0)	2(0.5)	0(0.0)	0(0.0)	0(0.0)	2(0.5)
Total	**59(15.4)**	**115(29.9)**	**145(37.8)**	**51(13.3)**	**10(2.6)**	**4(1.0)**	**384(100)**

## Discussion

In this study, the overall MDR rate of gram positive isolates (i.e. *S.aureus* and CNS) was 77%. This finding was slightly higher than 65.2% [13] and 52.7% [20] MDR rate documented for these two groups of bacteria in Ethiopia. But it is lower than 100% and 98.6% MDR reported by Mulu *et al.,* 2012 [21] and Biadglegne *et al.*, 2009 [22] in the same country respectively. The possible explanation for such disparity might be difference in study population where previous studies solely included hospitalized inpatients where higher MDR strains are expected. About 86.2% of *S.aureus* also became MDR of which 6.9% were resistant to all (ten) classes of antimicrobials tested. And again, 15.1% and 30.1% of them were resistant to seven and six classes respectively. Similarly, 28.6% of CNS showed MDR of which 21.4% were resistant to three classes (penicillin, tetracycline and phenicoles).

On the other hand, the overall MDR rate of gram negative bacteria tested for seven classes of antimicrobial drugs was 59.3%. This finding goes inline to study in Ethiopia where 51% MDR gram negative bacterial isolates from open wounds were reported (13). Moreover, the 100% MDR *Citrobacter* seen in this study concise with 100% MDR rate reported both in Ethiopia [22] and Pakistan [23], and 86.95% in Nepal [24]. Nearly 15% of *P.aeruginosa* was found to be resistant to 3 antimicrobial classes (Table 5) which is a bit higher than 10% report made by Kellen *et al.,*[7]*.* In that study, 15% of *K. pneumoniae* was reported as resistant to 3 antimicrobial classes which is higher than 6.5% obtained in the present study.

Regarding the resistance profile of isolates to individual drugs indicated that *S.aureus* showed an average resistance rate of 54.1% to most of the antimicrobial drugs tested (Table 3). This finding agrees with previous studies done elsewhere in Ethiopia [21,25-27] where average resistance of 52% up to 75% were recorded. About 76.7% of *S.aureus* was also oxacillin/methicillin resistant (MRSA). This finding was in agreement with findings in Ethiopia [28], Nepal [29], and Italy [30] where 83%, 60.6% and 74.2% were documented in that order. But, this was much lower than 100% resistant *S.aureus* to oxacillin reported by Yishak *et al.,* 2009 in Ethiopia [13]. And yet the 76.7% was incomparably higher than findings of Amare *et al.,* 2011 in Ethiopia [31], Anguzu *et al.,* in Uganda [12] and Wibbenmeyer *et al.,* 2006 in USA [32] where 34.6%, 25% and 46.2% MRSA were reported respectively. In this study, oxacillin resistant *S. aureus* (MRSA) were found out to be susceptible to cefoxitin, cefotaxime and ceftriaxone. The cause of oxacillin resistance in this case might not be because of macA gene instead, other mechanisms of resistance like impermeability of the membrane, deposition of high fat cover on cell wall, deformation/mutation of porine proteins extra could be reasons for such observed descripancies.

Moreover, the 16.4% vancomycin resistance rate of *S. aureus* in this study were lower than that of 40% reported by Mimejad *et al.,* 2008 in Iran [27] and 21% by Flamm *et al.,* 2004 in Nepal [29]. But, it was much higher when compared with 3.6% report made also in Iran [33]. However, such incidence of vancomycin resistant *Staphylococci* in hospital as well as in community are alarming because vancomycin is currently the main antimicrobial agent available to treat life-threatening infections with MRSA as indicated by CDC,2002 [34]. Unlike *S.aureus*, CNS was 100% sensitive for cefoxitin, erythromycin, clindamycin, norfloxacin, gentamycin, vancomycin, and ciprofloxacin. Similar high rate of susceptibility of CNS to these drugs were reported from Italy [30] and in Ethiopia (27).

Among gram negative isolates, *Proteus* species*, P.aeruginosa* and *Klebsiella* species showed high resistance (>65%) to doxycycline, cotrimoxazole and chloramphenicol. In a similar studies up to 100% resistance rate was reported in Ethiopia [9] and 83% in Pakistan [23].

In this study the most frequently isolated bacteria were *Proteus* species 107(27.9%) followed by *P.aeruginosa* 74(19.3%) and *S.aureus* 73(19%). The possible reason for the high frequency is that these bacteria are normal flora in healthy person when they get breaks on skins and soft tissue in any of mechanical cases or burns (especially *P.aeruginosa)* they can easily disseminate as it was indicated by Khanal *et al.,* 2010 in Nepal [29] and by Flamm *et al.,* in United States [35]. Moreover, these bacteria are commonly found in the hospital environment [8] which might increase wound infection rate and cross contamination among admitted patients.

## Conclusion

It is known that antimicrobial resistance is a growing global problem. However, the increased proportion of MDR seen in this study was considered as alarming because only a few treatment options remain for wound infections. About 76.7% of *S.aureus* was oxacillin/methicillin resistant (MRSA), of which 16.4% was vancomycin resistant (VRSA). Such incidence of vancomycin resistant *Staphylococci* is worrisome to the clinicians as it is currently the main antimicrobial agent available to treat life-threatening infections with MRSA. As majority of bacterial isolates showed widespread resistance against different antimicrobial classes, treatment of wound infections has to be made based on the culture and susceptibility results. Nevertheless, if one could not wait the culture results, ampicillin, penicillin, methicillin, trimethoprim-sulphamethoxazole, doxycyclin and chloramphenicol are not good choices to treat wound infections. Moreover, periodic monitoring of etiology and antimicrobial susceptibility of isolates from wounds in hospital settings is beneficial to the patient and assists physician in selection of chemotherapy in areas where no culture facilities.

## Competing interests

The authors declare that they have no competing interests.

## Authors’ contributions

GG, GK and HT participated in design, laboratory analysis, interpretation of the data and write up of the manuscript. All the authors read and approved the final version.
